# Locating Men in Sexual and Reproductive Health, Rights, and Justice: Past, Present, Futures

**DOI:** 10.1111/sifp.70003

**Published:** 2025-05-08

**Authors:** Joe Strong, Ernestina Coast, Malvern Chiweshe

## Abstract

Since the International Conference on Population and Development in 1994, global policies, and agenda‐setting milestones have emphasized that universal sexual and reproductive health and rights (SRHR) are unattainable without the meaningful engagement and inclusion of men. Despite this, the field of SRHR continues to struggle with how and in what ways men can and should be included in research, programs, and policies. In this commentary, we argue that the programmatic focus of SRHR limits the capacity to produce the data and evidence needed to inform gender transformational change. For men to be meaningfully engaged with by SRHR, researchers need an analytic lens that can capture the manifestations and outcomes of gender and power. We consider the conceptual complementarities between two theoretical frameworks: hegemonic masculinities and Reproductive Justice. We contend that bringing together these conceptual approaches to men and SRHR offers an analytic framework to iterate and innovate approaches to research. Such changes will allow for a greater interrogation of gender and power and the production of data and evidence necessary to grapple with the ongoing injustices that curtail people's sexual and reproductive freedoms.

## INTRODUCTION

Sexual and reproductive health and rights (SRHR) have a troubled relationship with men. Since the International Conference on Population and Development (ICPD) in 1994, global policies have acknowledged that universal SRHR cannot be achieved without grappling with and engaging men. Yet, the approaches used, and the progress made, have been inconsistent at best. To fulfill the goal of universal SRHR requires rethinking and reimagining how and in what ways we conceptualize and engage with the issues of gender and power in SRHR.

Men are critical actors in SRHR. Men dominate economic, political, cultural, and social institutions that can shape the legality, accessibility, and acceptability of SRHR (Chiweshe 2018; Starrs et al. 2018). Patriarchal systems of power have historically shaped health systems, leading to men's dominance in decision‐making processes. This includes what SRHR services are (legally) available and subsequently provided, how different populations are framed, and where the “problems” in SRHR are represented to be. Masculinist politics entrenches rigid gender norms that center on the control of women's bodies, while also propagating harmful myths of the infallibility (in health, at least) of men and boys. The same patriarchal structures have often excluded men from meaningful engagement in SRHR, particularly in ways that extend beyond their traditional roles as decision‐makers.

Failure to acknowledge men and boys in SRHR creates two linked challenges. First, it reduces the capability to design and implement services and reduces obstacles that men face when trying to exercise their rights and meet their health and well‐being needs (Porche 2012; Sonfield 2004; Maharaj 2000; Daniels 2006; Lohan 2015a; Strong 2024). Second, failure to consider the role of men in shaping the conditions that women and others face when navigating their SRHR constrains any attempt at transformational change, and universal progress (Greene and Biddlecom 2000; Kimport 2018). Men can be supportive of addressing the SRHR barriers that others face but can also be instrumental in re/producing those barriers (Freeman, Coast, and Murray 2017; Strong et al. 2022; Hook et al. 2018; Shand and Marcell 2021).

Renewed SRHR agendas and a WHO global priority‐setting exercise have emphasized the need to address the marginality of men in research (Brennan‐Wilson et al. 2024; Lohan et al. 2024). Despite the increasing awareness that gender transformational approaches are necessary for achieving universal SRHR (Ruane‐McAteer et al. 2020; Zielke et al. 2022; Brennan‐Wilson et al. 2024), a recent systematic review of male engagement in SRHR found that only 8 percent of current interventions with men use such approaches (Ruane‐McAteer et al. 2019). A 2024 review of 35 regional and global SRH policy documents found only one—the *WHO Strategy on the Health and well‐being of Men in the WHO Europe Region* (2018)—meaningfully connected equity of SRHR care for men and gender equality for all (Shand and Evoy 2024).

SRHR policies and programs require evidence that makes visible the components of gender power that need transforming. These issues make it imperative to engage more thoroughly with men and how men are conceptualized and incorporated into research. For gender transformational change to be realized, a shift in approaches to SRHR research is necessary. In this commentary, we outline how the field of SRHR has grappled with men post‐ICPD. We consider the theoretical, methodological, and empirical potential of greater critical engagement with the concepts of masculinities and Reproductive Justice for SRHR research and practice to more effectively engage with men.

## LOCATING MEN IN THE FIELD OF SRHR

ICPD took a rights‐based approach to shift the discourse around SRHR. The Program of Action argued for the need to incorporate the “key role” of men (United Nations Population Fund 2004, 28). Recommended outcomes in the program for action were focused on engaging men to achieve equality for women, emphasizing men's significant roles in shaping reproductive decision‐making and contraceptive use, fertility intentions, and conditions of parenting. This focus has been reflected in the development of SRHR‐related research in demography and allied disciplines.

The inclusion of men (and boys) in ICPD was a significant step forward. However, compromises between population control advocates and feminists meant that notions of overpopulation and a focus on the regulation of fertility, particularly of women racialized Black and Brown in the Global South, remained largely untroubled (Nandagiri 2021; Senderowicz and Valley 2023; Senderowicz 2020; Senderowicz and Nandagiri 2025). The focus on men's “responsibilities” in supporting women's “rights” (Basu 1996)—rights to access services and care that can support fertility regulation—illustrates the partial and inadequate ways that global SRHR policies have engaged with men. For example, it left little room to consider the SRHR needs and issues of men and boys nor unpacked the diversity of people within such broad population categories. In addition, there was little attention to unequal power dynamics, persistent gender imbalances, sociocultural barriers, and the lack of supportive societal structures.

Subsequent global policies—from Beyond ICPD 2014 to Sustainable Development Goal (SDG) Target 3.7^1^ and 5.6^2^—have retained a focus on women's contraceptive and SRHR decisions and policies, with no reference or indicators for measuring men's SRHR, nor gendered power imbalances and inequities (Wanner and Wadham 2015; High‐Level Task Force for ICPD 2013; UNDESA n.d.). These metrics reflect an agenda that is focused on women's bodies and has direct implications for the ways in which men are framed within the SRHR field (Greene and Biddlecom 2000). The resulting discourse uses the framing of “men as partners” to achieve these developmental aims (Wentzell and Inhorn 2014). Within this frame, the underlying aim of regulating women's fertility remains unchallenged and unchanged (Senderowicz and Nandagiri 2025).

The “men as partners” approach conceptualizes men as relevant because they can influence the SRHR of others (Inhorn et al. 2009; Culley, Hudson, and Lohan 2013). “Men as partners” is a prominent approach in SRHR policies and programs (Shand and Marcell 2021) with two major implications. First, men who are partnered with women become the population of interest, meaning that other men—such as those who are unpartnered, in unions that might not constitute “partnerships” under current definitions, and people who are trans/masculine or nonbinary—are made invisible. This is a key weakness in the approach, as other men represent a substantial population with their own SRHR needs and the capacity to influence the SRHR of others. Second, men become an instrument of “good”—and, therefore, a cause of “bad”—health and development outcomes in relation to women's reproduction. This contributes to men's “problematic” place in demography and SRHR more broadly (Greene and Biddlecom 2000).

The field of SRHR has (re)produced assumptions and created simplistic connections between complex and nuanced concepts. With (cis) women at the center of SRHR research, there is the assumption that a “gender” lens is either inherently employed or can be incorporated when a sample includes men. A comparison between two gender groups is important but is often unable to capture the ways in which gender is constructed through the interactions between people. Comparative analysis can tell us how two populations categorized by gender behave but not how their behavior is shaped by the gendered environment within which they live. For example, a comparison between genders can show couple discordance around contraceptive use, but not how and why this discordance emerges and the ways that it manifests in the interactions between people.

SRHR policies and programs are intertwined with SRHR research with men (Greene and Biddlecom 2000). A significant corpus of research on men and SRHR centers around contraceptive uptake and use. Men can use their gendered power to control decisions on reproduction while simultaneously expecting that women are responsible for achieving these decisions. Men can exert influence over women's contraceptive decisions, without women having the same influence in return (Ezeh 1993; Bankole 1995), including making the final decision regarding contraceptive use (Kabagenyi et al. 2014; Mbizvo and Adamchak 1991; Hartmann et al. 2016). Men might take ownership of the decision on which contraceptive to use while considering women responsible for contraceptive use (Hook et al. 2018; Hamm et al. 2019; Kabagenyi et al. 2014; Mbizvo and Adamchak 1991; Dral et al. 2018). Men have direct and indirect impacts on abortion‐related care, ranging from supporting to undermining women's decisions (Strong 2022; Freeman, Coast, and Murray 2017). Cultural and social contexts shape gendered expectations of masculinity and femininity, which in turn influence men's involvement in abortion‐related care—whether positively, negatively, or neutrally (Coast, Lattof, and Strong 2019; Strong 2022; Strong et al. 2022). Even when men do not appear to exert direct control, patriarchal norms embedded in cultural expectations can impact women's autonomy and decision‐making (Nartey, Bahar, and Nabunya 2023).

This existing work points to the important role that men can have in their partner's decision‐making and autonomy, but the constraints of internationally comparable data often limit further exploration of the role of gender and power in shaping these dynamics. Demographic research in particular is characterized by quantitative approaches using global, harmonized survey instruments (Blanc 2001) that mirror global agenda setting (Strong et al. 2023; Senderowicz 2020; Nandagiri 2021); the sample frames, methods, and SRHR issues centered on research reflect this agenda setting. These instruments often provide insights only for people in heterosexual, dyadic units, examining and comparing reported behaviors between men and women in such unions. These data cannot capture manifestations of gender occurring within and outside of these dyadic units, nor how power manifests and is enacted in context‐specific ways. Such evidence is needed to inform gender transformative policies and programs.

## CRITICAL THEORETICAL DEVELOPMENTS

The need to critically engage with key issues of gender and power within demographic research is well‐established (White 2000; Wanner and Wadham 2015), and the global goal of universal SRHR is undermined by insufficient attention to these issues. Measurements of reproductive agencies have operationalized a “can‐act‐resist” approach (Raj 2020; Raj et al. 2024). This aims to understand the external, as well as internal, factors that shape whether someone can make a decision with agency and includes making visible systems and structures of power. It is critical of the use of proxies such as contraceptive uptake or institution‐based childbirth (Raj 2020).

In this commentary, we propose a framework that complements this explicit analysis of power but focuses more explicitly on constructions of gender and the configurations of practice that form power hierarchies across and within populations. To do so, we identify two key theoretical developments that we suggest are essential to bring an analytic lens to SRHR: hegemonic masculinities and Reproductive Justice.

### Hegemonic Masculinities

Since the 1990s critiques around including men in SRHR center around resource scarcity and how program budgets designated for women and girls would be reduced, alongside concerns that including men would (re)entrench their power and presence in domains relating to women and girls (e.g., abortion) (Chant and Gutmann 2002; White 2000; Wanner and Wadham 2015). The analytic lens of hegemonic masculinities offers a way of tackling these criticisms and concerns (Mohr and Almeling 2020).

Masculinities refer to the “configurations of gender practice” with hegemonic masculinities being those configurations that most benefit from patriarchal structures (Connell 2005, 77). These configurations are reflected in the day‐to‐day behaviors, interactions, attitudes, and beliefs of people, wherein notions of “masculine” and “feminine” are constructed through particular practices.

A focus on masculinities, rather than men, requires centering how configurations of gender align to institutions, systems, and structures of power, connecting the individual and interpersonal with macro‐level systems. Sex, sexuality, and reproduction are nested within these gendered configurations, which offer an opportunity to include gender‐diverse and LGBTQ+ populations often excluded from research (Nyanzi 2011; Tamale 2011, 2014). Efforts to understand men and SRHR must effectively capture the macro‐level context within which men live. This allows for an analysis of how these systems shape and are shaped by gendered practices that can be interrogated to understand their function within SRHR expectations, attitudes, and behaviors. As Connell writes

Gender is social practice organised in relation to the reproductive arena, a process in which body‐reflexive practices are central. (Connell 1994, 14)

To understand masculinities, it is necessary for research to place people in their relational environments and consider how gender and power dynamics manifest through romantic, intimate, and sexual relationships and interactions with institutions and economic, social, cultural, and political systems (Levtov et al. 2014). The fundamentally constructed nature of these gendered configurations produces methodological challenges, particularly for quantitative, positivist approaches that are often unable to capture the nuances, complexities, and relationalities of gender dynamics beyond heterosexual couple dyads (Connell 2005).

Novel approaches to capturing masculinities and femininities have been tested, for example, Equimundo's Gender‐Equitable Men Scale (Levtov et al. 2014). Such scales can capture important evidence of men's SRHR practices and the attitudes and beliefs that drive them as well as methodological learnings for how future research may quantify the complexity of gender. Strong et al. (2022) evidence how men's attitudes and behaviors toward abortion‐related care are deeply relational, shaped by the relationship the man has with a woman, his own position within the community, and his ability to navigate racial capitalism economic systems. Abortion becomes, therefore, a site through which masculinities are enacted and practiced, severely curtailing the rights and freedoms of the person who is pregnant.

### Reproductive Justice

As the field of SRHR was being (re)defined in 1994, Reproductive Justice was being conceptualized in response to how ICPD framed SRHR and the individualism at the core of “choice”. Advocates, activists, scholars, and other experts—including those with lived experience—came together in the Black Feminist Collective Women of African Descent for Reproductive Justice and the SisterSong Women of Color Reproductive Health Collective (Ross and Solinger 2017a, 2017b).

Reproductive Justice advances the conceptualization of “rights” within international and national accords, requiring that rights be understood within social contexts of injustice, discrimination, and oppression (Gurr 2015). It provides a lens through which to interrogate individualizing “choice”‐based approaches to SRHR, seeking instead to center rights, and particularly access to SRHR‐related care within political and community locations, institutions, and structures (SisterSong, n.d.). Reproductive Justice challenges mainstream framings of SRHR to explicitly incorporate the gendered, racialized, and classed laws, policies, and environments that shape reproductive decision‐making (Ross and Solinger 2017a). Reproductive Justice is:

…a political movement that splices reproductive rights with social justice to achieve reproductive justice (Ross and Solinger 2017a, 9)

It rests on three critical tenets: (i) the right to have children, (ii) the right to not have children, and (iii) the right to parent in safe and sustainable environments. Reproductive Justice incorporates sexual autonomy and gender freedom as well as the right to bodily autonomy for all.

## TRANSFORMATIONAL FUTURES WITH NEW ANALYTIC APPROACHES

Research focused on operationalizing masculinities within SRHR has been useful in exposing gendered power dynamics (Mohr and Almeling 2020), for example, through the development of frameworks for understanding the gender politics of reproduction (Lohan 2015b), to men's own SRHR behaviors and health‐seeking (Shand and Marcell 2021), and to the gendered nature of health systems and service delivery (Dovel et al. 2020). Sexual and reproductive behaviors are gendered, which requires a theoretical lens that can capture the culturally sanctioned configurations of gender that shape SRHR expectations, practices, and outcomes (Atobrah 2017; Bhana 2016; Marston 2004). Masculinities offers a lens to more thoroughly consider how men shape the conditions that other people must navigate when trying to exercise their sexual and reproductive freedoms. For example, understanding the manifestation of masculinities in gendered notions of reproduction and parenting offers explanations for the ways in which men may seek to control pregnancies and abortion trajectories (Strong et al. 2022).

The concept of hegemonic masculinities can be expanded when used in conjunction with Reproductive Justice. Where interrogating hegemonic masculinities allows for an articulation of the ways that gendered hierarchies are constructed, incorporating Reproductive Justice then grounds analyses in how this shapes a person's rights and freedoms. Reproductive Justice is a tool through which to make visible the institutions and social practices that create oppressive environments and conditions that shape the people's freedoms to exercise their rights. As an analytic lens, it interrogates and shows the connections between systems, population policies and programs, and reproductive constraints and injustices (Nandagiri 2022). Reproductive Justice necessitates an intersectional approach that makes clear the ways in which (cis)gendered environments undermine and mire the sexual and reproductive experiences of people that challenge heteropatriarchal norms (Riggs and Bartholomaeus 2020; Riggs et al. 2020, 2021).

**FIGURE 1:**
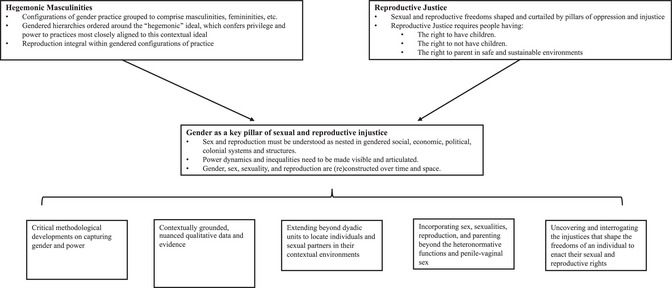
Hegemonic masculinities, Reproductive Justice, and their implications for research

There is limited research on men's rights to have children, not have children, or on their experiences of parenting in conditions of injustice. Using the theory of hegemonic masculinities together with the lens of Reproductive Justice lays bare the role of patriarchal institutions, structures, and systems in contributing to these injustices. Together, they articulate the manifestations of gendered practices and power dynamics that are critical to understand the constraints and restrictions on people's sexual and reproductive freedoms, and why policies and programs cannot have a transformational impact. Figure 1 conceptualizes how these frameworks create key requirements for researchers and considers some of the main implications for research, policy, and practice moving forward.

To unlock the full potential of using these complementary approaches, methodological approaches to understanding SRHR need to be inclusive of people regardless of their partnership or relationship status (Culley, Hudson, and Lohan 2013; Law 2019). Nuanced qualitative evidence is necessary for understanding the experiences of injustices and the ways in which gendered configurations of practice manifest within and between contexts; internationally comparable survey data are less able to capture the contextual specifics of gender and power dynamics. Survey scales and measurements that can capture components of gendered behaviors and practices offer opportunities for the development of surveys (Barker et al. 2011; Levtov et al. 2014; Edstrom et al. 2015). Constructing new, critical, and feminist quantitative research instruments that center modes of data collection to capture injustice, gender, and power will advance and expand the potential of SRHR research to inform policy and practice.

This includes being inclusive of broader genders and sexualities that much research continues to make invisible through its sampling frames; research on SRHR fundamentally fails where—gender—is taken as synonymous with cisgender women. Research in other social sciences—such as anthropology and sociology—has highlighted the importance of grappling with gender around issues such as infertility, andrology, and sperm (Dudgeon and Inhorn 2009; Inhorn 2020; Almeling 2020; Sigle 2021). Scholars and activists have shown the methodological and empirical need for more research on LGBTQ+ and gender‐diverse populations as a way of destabilizing and troubling normative, epistemological, and political assumptions (Riggs and Bartholomaeus 2020; Riggs et al. 2020; Westbrook, Budnick, and Saperstein 2021). Demographic research must engage with these spaces as relevant and forward‐facing or risk (re)producing out‐of‐date understandings of SRHR.

## CONCLUSIONS

Our commentary presents two complementary conceptual approaches that we argue are timely and necessary for researchers to incorporate into future work. Concurrently deploying masculinities and Reproductive Justice in sexual and reproductive research, particularly demographic research, invites us to consider novel methodologies that can enhance and nuance our empirical work. The resulting evidence will be foundational for gender transformational policies and programs that not only include men but also dismantle heteropatriarchal norms.

We offer some practical next steps as generative starting points for the field:

- *Further research on men's SRHR*: Key issues that impact many men's need to be made visible through research and evidence, including sexual violence, erectile dysfunction, male infertility, mental health and well‐being, prostate cancer, and Human papillomavirus (HPV). These are just some of the areas where men's SRH needs are made invisible.
- *Intersectional approaches*: Evidence and interventions need to ensure that men are not treated as a homogeneous population. Understanding the roles of disability, age, sexuality, class, race, and more provides the necessary intersectional lens to interrogate men's SRHR. This includes purposive research with populations made marginal, including gay, bisexual, and queer men, trans men, non‐binary folks, sex workers, men with disabilities, older men, adolescents, and unpartnered men.
- *Understanding health system barriers*: Further research needs to interrogate the gendered nature of health systems and the implications for men's access and health‐seeking behaviors, as well as how these systems (re)enforce gendered practices. This includes policies and clinical practice around Sexually Transmitted Infection (STI)‐related services, treatment of sexual violence, pregnancy care, health, and well‐being. How people experience these services and whether such care meets an individual's or community's needs is important.
- *Inclusive SRHR indicators*: National, regional, and global targets around SRHR need to develop indicators that incorporate men, gender, and power. This includes developing new approaches to measurement through surveys that allow for nuanced data around how power manifests across contexts.
- *Make visible systems and structures*: This includes the role of gender within social, economic, and political systems and policies and the implications these have for people's SRHR lives and freedoms, as well as the historical and cultural context of a population. This incorporates the analysis of policies around provision and funding toward programs.
- *Expanded research and development of male‐centered contraceptive methods*: Men's contraceptive options remain limited to the external condom and vasectomy. There is a need for enhanced research and development of additional non‐/hormonal male‐centered contraceptive methods. Examining the research and development of new SRHR technologies can expose how these (re)entrench contraception as gendered and a woman's burden by not prioritizing new methods for men.

These are not exhaustive but indicative of the gaps that can be addressed through using a combined lens of hegemonic masculinities and Reproductive Justice. These steps intersect across individual, interpersonal, community, and structural domains of health, allowing for work that meaningfully bridges the role that each domain has in shaping a person's SRHR. We hope for these can be prompts for further engagement with critical approaches to SRHR and gender within our field.

## Footnotes

1 SDG Target 3.7: Sexual and reproductive health: By 2030, ensure universal access to sexual and reproductive health‐care services, including family planning, information and education, and the integration of reproductive health into national strategies.

2 SDG Target 5.6: Ensure universal access to sexual and reproductive health and reproductive rights as agreed in accordance with the Programme of Action of the International Conference on Population and Development and the Beijing Platform for Action and the outcome documents of their review conferences
